# Horseradish peroxidase as an electrochemical reporter protein for cell-free biosensors

**DOI:** 10.1093/synbio/ysag009

**Published:** 2026-07-07

**Authors:** Aarushi Ruhela, Sahan B W Liyanagedera, Nadanai Laohakunakorn, Jamie R K Marland

**Affiliations:** School of Engineering, Institute for Integrated Micro and Nano Systems, University of Edinburgh, Edinburgh EH9 3FF, United Kingdom; Centre for Engineering Biology, Institute of Quantitative Biology, Biochemistry and Biotechnology, School of Biological Sciences, University of Edinburgh, Edinburgh EH9 3FF, United Kingdom; Biophoundry Inc, Chapel Hill, NC 27701, United States; Centre for Engineering Biology, Institute of Quantitative Biology, Biochemistry and Biotechnology, School of Biological Sciences, University of Edinburgh, Edinburgh EH9 3FF, United Kingdom; School of Engineering, Institute for Integrated Micro and Nano Systems, University of Edinburgh, Edinburgh EH9 3FF, United Kingdom

**Keywords:** cell-free biosensors, enzymatic reporter proteins, horseradish peroxidase, electrochemical readout, inducible gene expression

## Abstract

Cell-free biosensor systems offer a promising platform for portable diagnostics. Here, we evaluate electrochemical readout from these systems, using horseradish peroxidase (HRP) as a redox enzyme reporter. HRP was synthesized in an *Escherichia coli* cell-free transcription-translation system supplemented with hemin, calcium acetate, and commercial disulfide bond enhancers. Electrochemical detection of its activity was established by chronoamperometry, with hydrogen peroxide as a substrate and tetramethylbenzidine as a redox mediator. Cell-free expressed HRP produced a strong steady-state current compared to a catalytically inactive mutant and a no-template control. Kinetic analysis showed a *K_m_* for the cell-free expressed HRP close to that of the native enzyme. To explore the potential of HRP as an electrochemical reporter, we placed it under the control of a tetracycline-responsive regulatory promoter and demonstrated a 2.5-fold current increase in the presence of anhydrotetracycline. These results support HRP as an electrochemical reporter for cell-free biosensors, offering a complementary alternative to existing optical reporters for future use in handheld analytical devices.

## Graphical Abstract

**Graphical Abstract:**
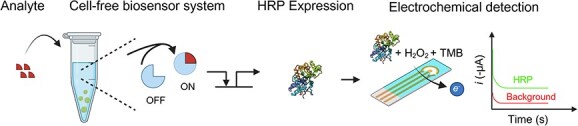

## Introduction

**Figure 1:**
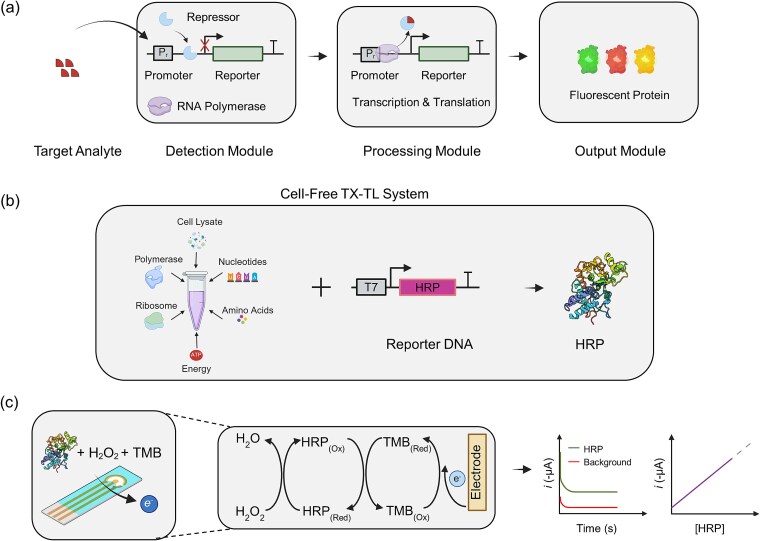
Schematic representation of the cell-free biosensor coupled with electrochemical detection using HRP as a reporter protein. (a) In the cell-free biosensor system, transcription of the reporter gene is repressed in the absence of the target analyte. Upon analyte binding, the repressor is displaced, activating transcription and leading to reporter expression. (b) Schematic representation of HRP expression from reporter DNA templates under a T7 promoter in an *Escherichia coli* cell-free TX-TL system. (c) The electrochemical detection mechanism relies on HRP-catalysed redox cycling of TMB in the presence of H_2_O_2_, producing a measurable current at the electrode surface. The output signal is recorded as a reduction current (i) over time, which increases proportionally with HRP concentration, enabling quantitative detection.

Biosensors are analytical devices that couple a biological recognition element with a physicochemical transducer, providing highly selective, sensitive, low-cost, and rapid readout. These advantages make them attractive for applications ranging from environmental monitoring to point-of-care diagnostics. Advances in synthetic biology have enabled new strategies for biosensor design and construction [1]. Among these, cell-free protein synthesis (CFPS) systems comprising cellular lysates or reconstituted transcription-translation (TX-TL) machinery enable *in vitro* gene expression without the constraints of cellular viability or regulation [2, 3]. CFPS platforms can therefore be engineered to detect target analytes and transduce this recognition into a measurable output signal [4]. Conceptually, cell-free biosensors consist of three functional modules: a detection module that recognizes the analyte, a processing module that transduces the recognition event, and an output module that generates a measurable signal (Fig. 1a) [5].

CFPS biosensor platforms commonly use reporters which provide an optical output that can be measured without physical contact [6]. Fluorescence can be read using laboratory-based plate readers, and recently portable field-deployable devices have also been demonstrated [7–11]. Colourimetric outputs can be measured by illuminating samples and detecting changes in brightness using individual sensors or plate-based camera systems [12, 13]. Electrochemical readout offers a potential complementary approach to these optical methods. Electrodes are readily miniaturized and compatible with compact inexpensive potentiostat instrumentation, similar to that used in personal glucose monitors. They also provide an inherently electronic readout which can be readily integrated into point-of-care diagnostics [14] and digital health platforms [15].

Electrochemical detection has been used extensively in the past to detect cells, proteins, nucleic acids, and small molecules [16] and, in some instances, redox enzymes have been employed to establish the interface between the biological events and electronic signals [17]. Enzyme-based reporters provide signal amplification due to their substrate turnover, enabling rapid readout and in some cases improved detectability [18]. Enzymes such as β-galactosidase and luciferase can be used to generate colourimetric or luminescent outputs [19, 20]. In this work, we explored horseradish peroxidase (HRP) as a redox enzyme reporter to create a quantifiable electronic output.

Native HRP is a glycosylated heme-containing monomeric protein of ~40 kDa in size, containing four disulfide bonds [21]. It has a well-established reagent chemistry with a broad range of readily available analytical tools and is widely used in biosensing and diagnostic applications for immunohistochemistry, enzyme-linked immunosorbent assays, *in-situ* hybridization, DNA biosensors and blot analysis [22]. Studies have shown that the recombinant expression of active HRP in *Escherichia coli* is possible but presents several challenges. It typically requires refolding steps or co-expression of folding chaperones and proper disulfide bond formation to restore enzymatic functionality [23–26], and the heme co-factor must be efficiently supplemented or biosynthesised for proper catalytic assembly [27]. Despite these challenges, active HRP can be expressed in CFPS by providing the necessary folding conditions and co-factors [28, 29].

In this work, we translated HRP in an *E. coli*-based cell-free TX-TL system (Fig. 1b). We further established amperometric detection for this cell-free expressed HRP using screen-printed gold electrodes (Fig. 1c). The expressed HRP catalyses the reduction of hydrogen peroxide (H_2_O_2_) to water, and oxidizes the mediator substrate, 3,3′,5,5′-tetramethylbenzidine (TMB), producing an electrochemically active product. The measured current output is generated from the reduction of HRP-oxidized TMB at the electrode surface, which increases proportionally with increasing HRP concentration. The resulting chronoamperometry (CA) response provides a quantitative electrochemical readout of analyte-dependent transcriptional activity (Fig. 1c). To evaluate HRP as a reporter for cell-free biosensors, we placed its expression under the control of the TetR/anhydrotetracycline (aTc) inducible regulatory system.

## Materials and methods

### DNA preparation

Synthetic gene fragments for the wild-type (WT) HRP, H170A mutant, T7tetOHRP and T7tetOmCherry were synthesized by Integrated DNA Technologies (sequences are reported in Supplementary Sequence Information S1) and assembled into the T7p14 expression vector using the Gibson Assembly® Master Mix [New England Biolabs (NEB)], following the manufacturer’s instructions. The resulting plasmid constructs were sequence-verified by Sanger sequencing (Genewiz) to confirm correct assembly. Subsequently, plasmid DNA was prepared using the ZymoPURE Plasmid Maxiprep Kit (Zymo Research) and linear DNA was PCR amplified using primers that bind 250 base-pairs upstream and downstream (Supplementary Table S1) of the inserts using Phusion™ High-Fidelity DNA Polymerase (NEB) following the manufacturer’s instructions. All the DNA samples were further purified using a DNA Clean & Concentrator-5 Kit (Zymo Research) to remove residual salts and enzyme contaminants before downstream applications.

### Crude extract preparation

Our procedure follows the protocol detailed fully in [30]. In brief, *E. coli* BL21 cell extracts were prepared using a mini-midi-maxi strategy, by growing cells in cultures of increasing size (5 ml, 50 ml, 400 ml) at 37°C to an OD_600_ of 1.8, before being pelleted, washed, and frozen at −80°C. The next day, the cell pellets were thawed, resuspended, and lysed with lysozyme (89833, Thermo Scientific) at a final concentration of 1 mg/ml for 90 min on ice. The 5 ml cell suspension was sonicated at 70% amplitude, 10 s on 10 s off, 1500 J using a 6 mm pre-cooled sonication probe (12931181, Fisher scientific) in an ice-water bath. The lysate was clarified by centrifugation at 12 000 × g, then subjected to an 80 min run-off reaction at 37°C before two more rounds of centrifugation at 30 000 × g. The clarified extract was dialysed overnight at 4°C against S30B buffer (14 mM magnesium glutamate, 150 mM potassium glutamate, adjusted to pH 8.2 using Tris) using a 10 kDa MWCO cassette (66380, Thermo Scientific). The final protein concentration was measured using a Bradford assay, and samples were snap frozen in 50 μl aliquots at 35 mg/ml and stored at −80°C until use.

### Cell-free TX-TL expression

The master mix for all the TX-TL reactions was made in a 1.5 ml microcentrifuge tube. Additive components were thawed on ice before adding to the tube. Cell-free TX-TL reactions of 10 μl volume were assembled with 10 nM plasmid or linear DNA and supplemented with appropriate concentrations of additives including nucleoside triphosphates, amino acids, transfer RNAs, salts, and crowding agents (Supplementary Table S2). The 3-phosphoglycerate-based energy solution (Supplementary Table S3) and amino acid solution (Supplementary Table S4) were prepared and used as previously described [31]. For HRP expression, reaction mixtures were supplemented with 15 μM hemin, 0.5 mM calcium acetate, 2 mM magnesium glutamate, and a commercial disulfide bond enhancer kit (PURExpress® Disulfide Bond Enhancer, NEB) to facilitate proper folding and catalytic activity of the HRP enzyme. Reactions were incubated in PCR tubes at 20°C for 8 h using a thermocycler (ProFlex PCR System). For mCherry, magnesium glutamate was added to a final concentration of 8 mM, and reactions were incubated in a 384-well plate at 29°C for 12 h using a plate reader (Biotek Synergy H1). Reactions with tet-regulated promoter templates were supplemented with 100 nM of recombinant TetR protein (HY-P71520, MedChem) and/or 1 μM of anhydrotetracycline hydrochloride (94 664, Merck).

### Optical detection of HRP activity

The activity of the expressed HRP was confirmed using a conventional colourimetric assay based on the oxidation of TMB in the presence of H_2_O_2_. Briefly, 90 μl of commercial TMB substrate solution (T4444, Merck) was mixed with 10 μl of diluted cell-free reaction product and incubated for 10 min at room temperature, followed by quenching with 50 μl of 1 M H_2_SO_4_. Absorbance was measured at 450 nm using a FLUOstar Omega plate reader (BMG Labtech). Blank absorbance from substrate-only controls was subtracted from sample readings prior to data analysis.

### Electrochemical detection of HRP activity

Electrochemical measurements were performed using an EmStat3 Blue or Palmsens4 potentiostat (PalmSens BV) with commercial screen-printed electrodes (DRP-C223BT-U75, DropSens) consisting of a 1.6 mm diameter of gold working electrode, silver reference electrode, and gold counter electrode. All potentials are reported relative to the Ag pseudo-reference electrode. Electrochemical cleaning of the electrode surface was performed using 0.1 M H_2_SO_4_ with CV carried out at a scan rate of 0.1 V/s with a step potential of 0.002 V over a range of 0.0–1.6 V before taking any further readings. Later, CV was carried with 40 μl of samples of interest at a scan rate of 0.1 V/s with a step potential of 0.005 V, over a range of −0.2 to +0.1 V. TMB substrate (T4444 or T3405, Merck) was supplemented with 10 mM KCl, and native HRP (P8375, Merck) was used for these initial experiments. CA was performed at an applied potential of 0.0 V for 60 s with a sampling interval of 0.1 s, and the steady-state current (averaged current readings from 50 to 60 s) was evaluated.

### Fluorescence detection of cell-free reporters

For mCherry expression, 10 μl reactions were set up in a black 384-well plate and incubated at 29°C for 12 h in a Biotek Synergy H1 plate reader, and the signal was read every 2 min with orbital shaking using excitation at 579 nm and emission at 616 nm with a gain setting of 50.

### Statistical analysis

Data were analysed using GraphPad Prism version 11.0.1. Error bars represent the standard error of the mean (SEM). Statistical significance was assessed using an unpaired two-tailed T-test (for comparison of two conditions) or Analysis of Variance followed by Bonferroni post-hoc tests (for comparison of three or more conditions). Non-linear regression analyses were performed using the Michaelis-Menten model for enzyme kinetics and the Gompertz model for TX-TL kinetics of HRP and mCherry.

## Results

### Amperometric detection of HRP

**Figure 2:**
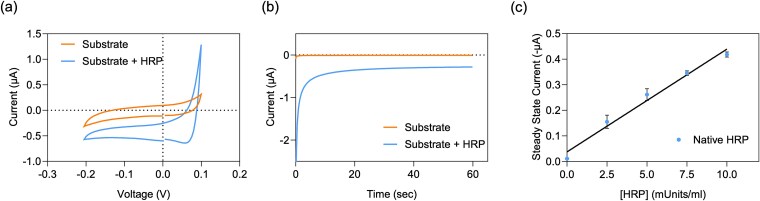
(a) Cyclic voltammetry of substrate solution without and with HRP (5 mUnits/ml), recorded over a potential range of −0.2 to +0.1 V. (b) CA of substrate solution without and with HRP (5 mUnits/ml), performed at an applied potential of 0.0 V for 60 s (c) Calibration curve of steady-state CA current as a function of HRP concentration (*n* = 3 replicates), showing a linear response (*R*^2^ = 0.95). Calibration was performed under the same CA conditions. All measurements were taken after a 2-min incubation of HRP with substrate prior to electrochemical readings.

We first established an electrochemical protocol for HRP detection based on the HRP-mediated generation of oxidized TMB (TMB_ox_), which is subsequently reduced at the electrode. Purified native HRP was used in these initial experiments to validate the protocol before applying it to the HRP expressed from the cell-free system. Cyclic voltammetry of the substrate solution containing H_2_O_2_ and TMB was performed to characterize the system. The anodic sweep was limited to +0.1 V to avoid direct electrochemical oxidation of TMB at the electrode. Substrate alone showed a negligible Faradaic reduction current, consistent with the absence of TMB_ox_ (Fig. 2a). In contrast, incubation of the substrate with HRP (5 mUnits/ml) for 2 min produced a pronounced reduction current, with a cathodic peak at approximately +0.06 V, corresponding to the reduction of enzymatically oxidized TMB (Fig. 2a). The highest signal-to-background separation occurred at 0.0 V, which was therefore selected for CA to quantify HRP activity. CA measurements showed an initial transient current, followed by the development of a steady-state reduction current. The steady-state current of substrate-only control was −8.3 ± 1.1 nA, whereas the presence of HRP (5 mUnits/ml) increased the current to −302.6 ± 25.9 nA, confirming that the CA signal reflected HRP activity (Fig. 2b).

Then, to determine the sensitivity and LoD of the assay, steady-state CA currents were measured at varying concentrations of HRP. As shown in Fig. 2c, a clear linear relationship between HRP concentration and steady-state current was observed, enabling quantitative sensing. The sensitivity to HRP was −40.17 ± 2.43 nA per (mUnit/ml) with an offset of −0.04 ± 0.01 μA. The LoD (three times the standard deviation of the blank signal, divided by sensitivity) was 0.14 mUnits/ml.

### Cell-free expression and electrochemical detection of HRP

**Figure 3:**
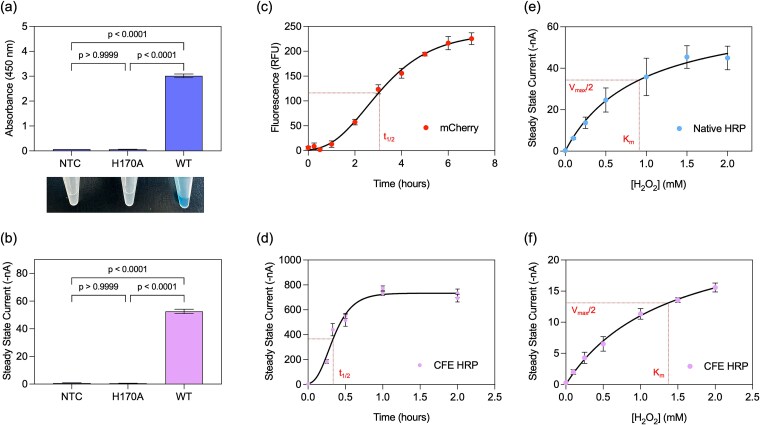
Cell-free reactions were assembled with 10 nM DNA template encoding WT or the catalytically inactive mutant H170A; NTC served as negative controls. (a) Spectrophotometric detection of HRP activity using TMB substrate for NTC, H170A, and WT, with photographs of the corresponding reaction tubes shown below. (b) Electrochemical detection using chronoamperometric steady-state current for NTC, H170A, and WT reactions. (c) Time-course of mCherry fluorescence used as a TX-TL reporter, with the half-maximum time (t_1/2_) indicated (*n* = 3 replicates). (d) Time-course of steady-state chronoamperometric current for HRP expression in the cell-free system, with t_1/2_ indicated (two experimental runs per timepoint, with runs plotted separately; each point represents the mean from *n* = 3 replicates). Michaelis–Menten analysis of (e) purified native HRP (4 mUnits/ml) and (f) CFE HRP (10 μl of 10× diluted reaction product), determined from steady-state chronoamperometric currents at varying H_2_O_2_ concentrations and fixed TMB (200 μM) (*n* = 3 replicates for each condition).

Expression of HRP in the cell-free TX-TL system was first optimized by varying the incubation temperature (Supplementary Fig. S1), and concentrations of hemin, calcium, and magnesium (Supplementary Fig. S2), and screened for the effect of commercial disulfide bond enhancers (Supplementary Fig. S3). The cell-free expression and functional validation of HRP were initially assessed using an optical assay with H_2_O_2_ and TMB as a chromogenic substrate. In the presence of HRP, TMB is converted into a coloured product with absorbance at 450 nm when quenched with acid. A significant increase in absorbance was observed in the WT HRP expressing reaction, whereas negligible signals were detected in the no DNA template control (NTC) or a catalytically inactive mutant (H170A) [32] (Fig. 3a). Absorbance readings at 450 nm for HRP (3.014 ± 0.074) were significantly greater than those of NTC (0.064 ± 0.001) or H170A (0.066 ± 0.002). After confirming HRP expression using the optical method, we next evaluated the electrochemical detection of cell-free expressed HRP. The steady-state reduction current was markedly larger in the WT HRP sample (−52.56 ± 1.58 nA) compared to both the NTC (−0.73 ± 0.13 nA) and the H170 mutant (−0.55 ± 0.06 nA), confirming that the observed signal originated from the enzymatic activity of HRP (Fig. 3b). No significant difference was observed between NTC and H170A in both optical and electrochemical methods, indicating that the signal was specific to the functional peroxidase and not due to background activity from the cell-free extract, and that the H170A variant lacks measurable enzymatic activity. SDS-PAGE showed successful translation of both H170 and WT HRP (Supplementary Fig. S4). Furthermore, the electrochemical results were consistent with those obtained from the optical assay, demonstrating the reliability of the electrochemical platform in detecting cell-free expressed HRP.

Previous work has shown that the enzymatic reporters can mature more rapidly than fluorescent protein reporters in cell-free systems [18]. To explore this, we measured the expression kinetics of HRP and the fluorescent protein mCherry. For HRP measurements, individual cell-free reaction tubes were removed at defined time intervals during incubation, snap frozen, then thawed and analysed together by CA. Separate mCherry expressing reactions were incubated in a plate reader, with fluorescence recorded from the same wells over time. Reporter outputs were fitted using an asymmetric sigmoidal growth model (Gompertz) that captures both the initial lag phase and the eventual saturation. The time to reach half the maximum signal was estimated to be 20 min for HRP, compared with 184 min for mCherry (Fig. 3c and d). This result is consistent with the previous reports of enzymatic reporters providing substantially faster readout than fluorescent proteins, and is advantageous for rapid signal generation in biosensing applications.

Finally, to assess the functional quality of cell-free expressed HRP, we performed a Michaelis–Menten kinetic analysis using native HRP and cell-free expressed HRP (Fig. 3e). The steady-state currents were recorded at varying H_2_O_2_ concentrations with a fixed concentration of TMB. For native HRP, the resulting curve showed characteristic saturating behaviour, with an apparent *K_m_* of 0.91 ± 0.96 mM. The cell-free expressed HRP displayed a comparable kinetic profile, yielding a *K_m_* of 1.37 ± 0.48 mM (Fig. 3f).

### Electrochemical detection of HRP as a reporter protein

**Figure 4:**
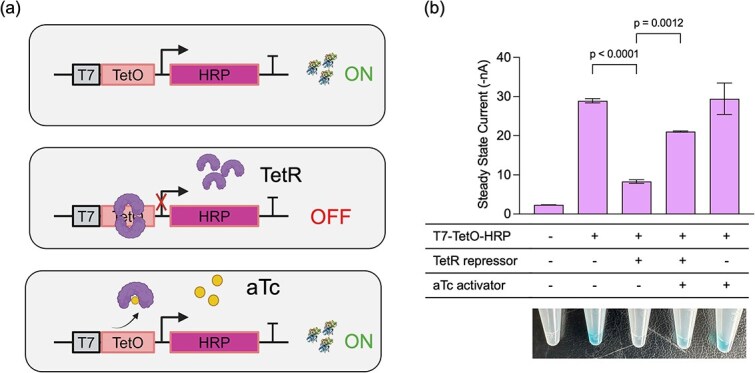
(a) Schematic of the TetR-regulated HRP reporter system. In the absence of TetR, HRP is expressed (ON state). The binding of TetR to the TetO operator represses transcription (OFF state). The addition of aTc inhibits TetR, restoring HRP expression (ON state). (b) Electrochemical detection of HRP activity in the cell-free system, where reactions were set up with 10 nM DNA and/or 100 nM TetR and/or 1 μM aTc (*n* = 3 replicates for each condition). A photograph of colourimetric TMB oxidation (bottom) and the steady-state CA currents (top) are shown.

Finally, as a proof-of-principle demonstration that HRP can function as a reporter protein from a cell-free biosensor system, its expression was placed under the control of a regulated promoter. A commonly used tetracycline-inducible system was selected to validate whether modulation of gene expression can be reliably transduced into a measurable electrochemical signal [11, 33, 34]. The tet-operator (tetO) sequence was positioned downstream of a T7 promoter driving the expression of HRP. Recombinant TetR protein that recognizes and binds the tetO site was added to the cell-free reaction, and it represses basal HRP transcription. Binding of the inducer aTc to TetR releases it from the tetO site, thereby permitting HRP expression (Fig. 4a). Cell-free reactions were prepared with the addition of TetR and/or aTc. Initially, to optimize the template design, mCherry expression was regulated with this promoter and showed a 4.5-fold difference between the repressed (OFF) and aTc induced (ON) states (Supplementary Fig. S5). The system was then implemented with HRP as a reporter. As shown in Fig. 4b, CA measurements revealed a reduction current of −8.31 ± 0.45 μA in the absence of aTc, while a 2.5-fold larger current of −21.06 ± 0.16 μA was measured upon induction, indicating active HRP expression (Fig. 4b). A similar 2.1-fold increase in absorbance was observed from the colourimetric readout (Supplementary Fig. S6). Increasing aTc concentrations led to a dose-dependent induction of HRP expression (Supplementary Fig. S7). Together, these results confirm that the synthetic regulatory promoter functioned as intended and that HRP expression was regulated by the tetO system. The successful chronoamperometric detection of HRP activity demonstrates that HRP functions effectively as an electrochemical reporter in the cell-free TX-TL systems, providing a direct and quantifiable connection between the sensing input and the electrical output.

## Discussion

The goal of this work was to combine a cell-free TX-TL biosensor system with an electrochemical output using HRP as a reporter protein. This may increase the utility of such biosensor systems for deployment in practical applications.

In this study, HRP was successfully expressed in a CFPS system, consistent with previous work by Zhu *et al.* and Park *et al.*, who demonstrated the functional expression of HRP in a cell-free platform [28, 29]. Similar optimal expression conditions of 20°C temperature, 15 μM hemin were observed in our work, whereas the optimal Ca^2+^ concentration was found to be lower at 0.5 mM when optimized alongside Mg^2+^ (Supplementary Fig. S1 and S2). Importantly, the *K_m_* observed from cell-free expressed HRP (1.37 ± 0.48 mM) was similar to the native HRP (0.91 ± 0.96 mM), and consistent with reported literature values of 0.7 ± 0.2 mM [35]. The close agreement between these values confirms that the cell-free product is catalytically active and maintains substrate affinity similar to the purified enzyme. Although native glycosylation, which supports folding, solubility, and stability [36], is absent in HRP expressed in the *E. coli* cell-free system, it maintains similar kinetic behaviour. These findings advance the utility of HRP as a reporter enzyme for cell-free biosensing applications.

CFPS platforms offer various advantages over conventional methods used for biosensing. However, they also have limitations. The stability of cell-free reactions outside laboratory settings has been a challenge and can be mitigated by employing freeze-dried extracts, as demonstrated by Pardee *et al.* [33]. The optimal HRP expression temperature of 20°C gives rise to a practical limitation for integrating HRP into more complex cell-free circuits, since other sensing or regulatory modules may show optimal translation or function at higher temperatures. However, this is not expected to fundamentally affect the utility of HRP, as other circuit components could be pre-expressed directly in source cells prior to lysate preparation, or added as purified proteins if necessary. Operation near room temperature may also be advantageous for real-world deployment, since it could reduce reliance on controlled incubators and may better suit field or point-of-care settings.

Electrochemical output modules from cell-free TXTL biosensor systems are relatively underexplored. However, in addition to this work, some other approaches have been demonstrated previously. One such example is the use of a nucleic acid-based output module, which relies on synthetic DNA tagged with a redox label such as methylene blue or ferrocene, detected using pulsed amperometry. Tagged single-stranded DNA may be immobilized at an electrode, enabling detection of RNA transcribed within the system and bound to the labelled probe [37]. Alternatively, untagged single-stranded DNA may be immobilized at the electrode and can capture tagged DNA following its release by translated restriction enzymes [34]. While these readout methods offer high specificity, they require costly tagged DNA and lack the amplification provided by enzyme-based output modules.

Other approaches have interfaced cell-free systems with personal glucose meters by expressing reporter enzymes such as invertase, trehalase, or β-galactosidase, which generate glucose as a measurable product [38–41]. This approach enables enzymatic signal amplification using well-established and inexpensive readout technology, but it is vulnerable to distorted measurements due to reaction mixture metabolic activity (such as glycolysis) and existing glucose within test samples. Use of HRP as an electrochemical enzymatic reporter may avoid these potential sources of interference and allow direct redox coupling between the expressed reporter and the electrode. However, enzymatic signal amplification also has the drawback that it may amplify the effects of leaky reporter expression, which can increase the OFF-state background and reduce dynamic range [42]. To mitigate this, future biosensor circuits with HRP readout may require optimization of promoter strength, reaction timing, and mediator/substrate conditions to balance rapid signal generation against background.

Looking forward, the work presented is well-positioned for integration with a broad range of biosensing applications such as biomedical diagnostics and environmental monitoring [43]. HRP could be used with a wide range of cell-free sensor modules that trigger reporter protein translation, including riboswitches, toehold switches, allosteric transcription factors, and nanobody receptors [6, 44]. There is also scope in exploring alternative redox reporters or more stable substrates other than TMB to broaden the toolkit available for cell-free biosensors, offering new avenues for optimizing both sensitivity and operational stability [35].

## Conclusion

The HRP-based electrochemical reporter system described here combines an enzymatic reporter with direct electrochemical readout, offering a complementary alternative output for CFPS biosensors. We believe that this work will open new opportunities to expand the use of CFPS for on-site biosensing and diagnostics. With the increasing integration of bioelectronics, this study also bridges the gap between synthetic biology and electronics, providing a foundation for the future development of lab-on-chip electronic devices.

## Supplementary Material

Supplementary-material_ysag009

## Data Availability

The data underlying this article is available on request.

## Materials availability

The sequences for the DNA used in this work are given in the Supplementary Sequence Information file.

## References

[1] Nielsen AAK, der BS, Shin J et al. Genetic circuit design automation. Science 2016;352:aac7341. 10.1126/science.aac7341 27034378

[2] Hodgman CE, Jewett MC. Cell-free synthetic biology: thinking outside the cell. Metab Eng 2012;14:261–9. 10.1016/j.ymben.2011.09.002 21946161 PMC3322310

[3] Carlson ED, Gan R, Hodgman CE et al. Cell-free protein synthesis: applications come of age. Biotechnol Adv 2012;30:1185–94. 10.1016/j.biotechadv.2011.09.016 22008973 PMC4038126

[4] Lee P-W, Maerkl SJ. Regulatory components for bacterial cell-free systems engineering. ACS Synth Biol 2024;13:3827–41. 10.1021/acssynbio.4c00574 39509282 PMC11669159

[5] Zhang L, Guo W, Lu Y. Advances in cell-free biosensors: principle, mechanism, and applications. Biotechnol J 2020;15:2000187. 10.1002/biot.202000187 32667120

[6] Arce A, Brown DM, Demissie HA et al. Cell-free biosensors: where have we been and where do we need to go? Curr Opin Biotechnol 2026;98:103451. 10.1016/j.copbio.2026.103451 41671650

[7] Gräwe A, Dreyer A, Vornholt T et al. A paper-based, cell-free biosensor system for the detection of heavy metals and date rape drugs. PLoS One 2019;14:e0210940. 10.1371/journal.pone.0210940 30840628 PMC6402643

[8] Ma Y, Mou Q, Yan P et al. A highly sensitive and selective fluoride sensor based on a riboswitch-regulated transcription coupled with CRISPR-Cas13a tandem reaction. Chem Sci 2021;12:11740–7. 10.1039/d1sc03508h 34659710 PMC8442723

[9] Mahas A, Wang Q, Marsic T et al. Development of Cas12a-based cell-free small-molecule biosensors via allosteric regulation of CRISPR array expression. Anal Chem 2022;94:4617–26. 10.1021/acs.analchem.1c04332 35266687 PMC8943526

[10] Misteli T et al. qByte: an open-source isothermal fluorimeter for democratizing analysis of nucleic acids, proteins and cells. PLoS Biol 2025;23:e3003199. 10.1371/journal.pbio.3003199 PMC12157786 40435312

[11] Jung JK, Alam KK, Verosloff MS et al. Cell-free biosensors for rapid detection of water contaminants. Nat Biotechnol 2020;38:1451–9. 10.1038/s41587-020-0571-7 32632301 PMC7718425

[12] Karlikow M, da Silva SJR, Guo Y et al. Field validation of the performance of paper-based tests for the detection of the Zika and chikungunya viruses in serum samples. Nat Biomed Eng 2022;6:246–56. 10.1038/s41551-022-00850-0 35256758 PMC8940623

[13] Pardee K, Green AA, Takahashi MK et al. Rapid, low-cost detection of Zika virus using programmable biomolecular components. Cell 2016;165:1255–66. 10.1016/j.cell.2016.04.059 27160350

[14] Wachholz Junior D, Deroco PB, Hryniewicz BM et al. Strategies for electrochemical point-of-care biosensors. Annu Rev Anal Chem 2025;18:307–33. 10.1146/annurev-anchem-071124-103739 40014647

[15] Wu J, Liu H, Chen W et al. Device integration of electrochemical biosensors. Nat Rev Bioeng 2023;1:346–60. 10.1038/s44222-023-00032-w 37168735 PMC9951169

[16] Lin M, Song P, Zhou G et al. Electrochemical detection of nucleic acids, proteins, small molecules and cells using a DNA-nanostructure-based universal biosensing platform. Nat Protoc 2016;11:1244–63. 10.1038/nprot.2016.071 27310264

[17] Wen W, Yan X, Zhu C et al. Recent advances in electrochemical immunosensors. Anal Chem 2016;89:138–56. 10.1021/acs.analchem.6b04281 28105820

[18] Lopreside A, Wan X, Michelini E et al. Comprehensive profiling of diverse genetic reporters with application to whole-cell and cell-free biosensors. Anal Chem 2019;91:15284–92. 10.1021/acs.analchem.9b04444 31690077 PMC6899433

[19] Pellinen T, Huovinen T, Karp M. A cell-free biosensor for the detection of transcriptional inducers using firefly luciferase as a reporter. Anal Biochem 2004;330:52–7. 10.1016/j.ab.2004.03.064 15183761

[20] Hunt JP, Free TJ, Galiardi J et al. Streamlining the detection of human thyroid receptor ligand interactions with XL1-blue cell-free protein synthesis and beta-galactosidase fusion protein biosensors. Life 2023;13:1972. 10.3390/life13101972 PMC10608756 37895354

[21] Lavery CB, MacInnis MC, MacDonald MJ et al. Purification of peroxidase from horseradish (*Armoracia rusticana*) roots. J Agric Food Chem 2010;58:8471–6. 10.1021/jf100786h 20681636

[22] Lopes GR, Pinto DCGA, Silva AMS. Horseradish peroxidase (HRP) as a tool in green chemistry. RSC Adv 2014;4:37244–65. 10.1039/c4ra06094f

[23] Ortlepp SA, Pollard-Knight D, Chiswell DJ. Expression and characterisation of a protein specified by a synthetic horseradish peroxidase gene in *Escherichia coli*. J Biotechnol 1989;11:353–64. 10.1016/0168-1656(89)90019-9

[24] Veitch NC . Horseradish peroxidase: a modern view of a classic enzyme. Phytochemistry 2004;65:249–59. 10.1016/j.phytochem.2003.10.022 14751298

[25] Smith AT, Santama N, Dacey S et al. Expression of a synthetic gene for horseradish peroxidase C in *Escherichia coli* and folding and activation of the recombinant enzyme with Ca2+ and heme. J Biol Chem 1990;265:13335–43. 10.1016/s0021-9258(19)38303-6 2198290

[26] Bartonek-Roxå E, Eriksson H. Expression of a neutral horseradish peroxidase in *Escherichia coli*. J Biotechnol 1994;37:133–42. 10.1016/0168-1656(94)90004-3 7765453

[27] Gazaryan JG, Doseeva VV, Galkin AG et al. Effect of a negative charge on the screening of the active site of horseradish peroxidase. Russ Chem Bull 1995;44:363–6. 10.1007/bf00702153

[28] Park Y-J, Kim D-M. Production of recombinant horseradish peroxidase in an engineered cell-free protein synthesis system. Front Bioeng Biotechnol 2021;9:778496. 10.3389/fbioe.2021.778496 PMC8579056 34778239

[29] Zhu B, Mizoguchi T, Kojima T et al. Ultra-high-throughput screening of an in vitro-synthesized horseradish peroxidase displayed on microbeads using cell sorter. PLoS One 2015;10:e0127479. 10.1371/journal.pone.0127479 PMC4439038 25993095

[30] Liyanagedera SBW, Williams J, Wheatley JP et al. SpyPhage: a cell-free TXTL platform for rapid engineering of targeted phage therapies. ACS Synth Biol 2022;11:3330–42. 10.1021/acssynbio.2c00244 36194543

[31] Sun ZZ, Hayes CA, Shin J et al. Protocols for implementing an *Escherichia coli* based TX-TL cell-free expression system for synthetic biology. J Vis Exp 2013;79:e50762. 10.3791/50762 PMC3960857 24084388

[32] Newmyer SL, Sun J, Loehr TM et al. Rescue of the horseradish peroxidase His-170 → ala mutant activity by imidazole: importance of proximal ligand tethering. Biochemistry 1996;35:12788–95. 10.1021/bi9609331 8841121

[33] Pardee K, Green AA, Ferrante T et al. Paper-based synthetic gene networks. Cell 2014;159:940–54. 10.1016/j.cell.2014.10.004 25417167 PMC4243060

[34] Sadat Mousavi P, Smith SJ, Chen JB et al. A multiplexed, electrochemical interface for gene-circuit-based sensors. Nat Chem 2019;12:48–55. 10.1038/s41557-019-0366-y 31767994 PMC7700015

[35] Kergaravat SV, Pividori MI, Hernandez SR. Evaluation of seven cosubstrates in the quantification of horseradish peroxidase enzyme by square wave voltammetry. Talanta 2012;88:468–76. 10.1016/j.talanta.2011.11.016 22265528

[36] Asad S, Khajeh K, Ghaemi N. Investigating the structural and functional effects of mutating Asn glycosylation sites of horseradish peroxidase to Asp. Appl Biochem Biotechnol 2010;164:454–63. 10.1007/s12010-010-9147-1 21193964

[37] Bracaglia S, Ranallo S, Ricci F. Electrochemical cell-free biosensors for antibody detection. Angew Chem Int Ed 2023;62:e202216512. 10.1002/anie.202216512 36533529

[38] Lee PW, Mottaghi SS, Lugnier MG et al. A T7 RNAP regulatory toolbox for cell-free network engineering and biosensing applications. Nat Commun 2026;17:6941. 10.1038/s41467-026-73811-9 PMC13389190 42209525

[39] Zhang Y, Steppe PL, Kazman MW et al. Point-of-care analyte quantification and digital readout via lysate-based cell-free biosensors interfaced with personal glucose monitors. ACS Synth Biol 2021;10:2862–9. 10.1021/acssynbio.1c00282 34672518 PMC9807263

[40] Amalfitano E, Karlikow M, Norouzi M et al. A glucose meter interface for point-of-care gene circuit-based diagnostics. Nat Commun 2021;12:724. 10.1038/s41467-020-20639-6 PMC7851131 33526784

[41] Jang Y-J, Lee K-H, Yoo TH et al. Interfacing a personal glucose meter with cell-free protein synthesis for rapid analysis of amino acids. Anal Chem 2019;91:2531–5. 10.1021/acs.analchem.8b05526 30667232

[42] Hicks M, Bachmann TT, Wang B. Synthetic biology enables programmable cell-based biosensors. ChemPhysChem 2019;21:132–44. 10.1002/cphc.201900739 31585026 PMC7004036

[43] Green TP, Talley JP, Bundy BC. Recent advances in developing cell-free protein synthesis biosensors for medical diagnostics and environmental monitoring. Biosensors 2025;15:499. 10.3390/bios15080499 PMC12385134 40862960

[44] Lee K-H, Kim D-M. In vitro use of cellular synthetic machinery for biosensing applications. Front Pharmacol 2019;10:1166. 10.3389/fphar.2019.01166 PMC6803485 31680954

