# Supplementary material for: Horseradish peroxidase as an electrochemical reporter protein for cell-free biosensors
Source: Synth Biol (Oxf). 2026 Jul 7;11(1):ysag009. doi: 10.1093/synbio/ysag009 (PMC13455610; doi:10.1093/synbio/ysag009)
Supplement: Supplementary file 1 [file supplementary-material_ysag009.zip › 2026-05-29 Supplementary Information_ysaf009.pdf]

Supplementary Information
Horseradish Peroxidase as an electrochemical reporter protein for cell-free biosensor systems

Aarushi Ruhela, Sahan B. W. Liyanagedera, Nadanai Laohakunakorn, Jamie R. K. Marland

1 Supplementary Figures

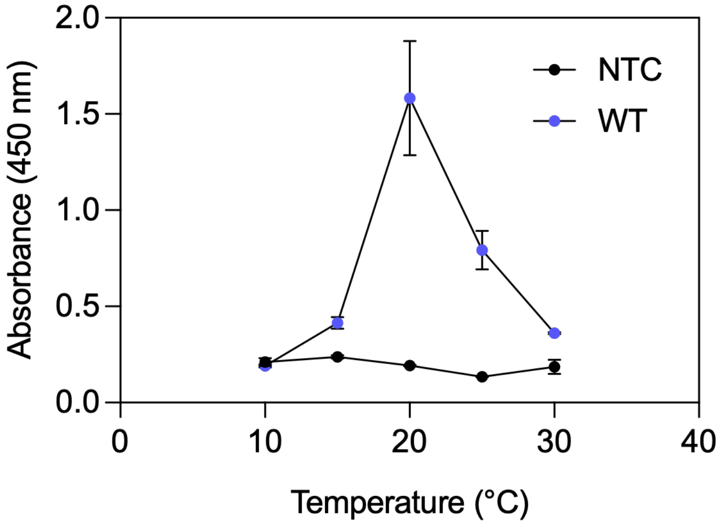

Figure S1: **Temperature optimisation of HRP expression in the cell-free system.** HRP activity was measured at different incubation temperatures by spectrophotometric detection of TMB oxidation. Wild-type HRP (WT, blue) shows maximal activity at 20 °C, whereas the no-template control (NTC, black) exhibits negligible signal across all tested temperatures (n = 3 replicates for each condition).

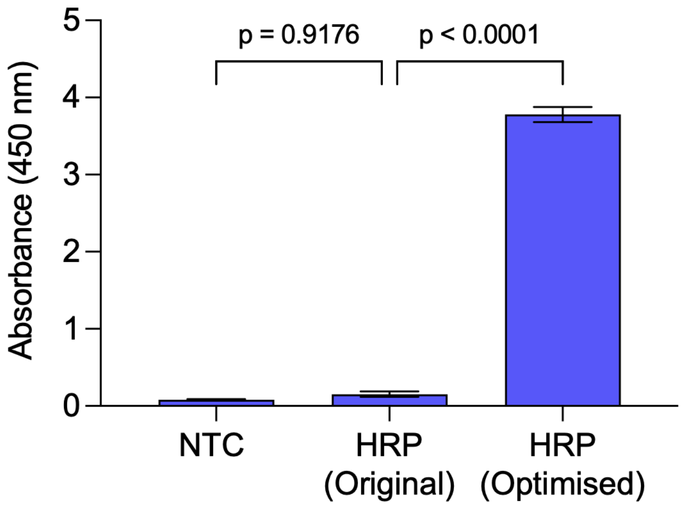

Figure S2: **Optimisation of cofactors for HRP expression in the cell-free system.** HRP was expressed at its optimal incubation temperature (20 °C) under different cofactor conditions and quantified by spectrophotometric detection of TMB oxidation at 450 nm following a 500-fold dilution of reaction products. "HRP (Original)" indicates the conditions before optimisation (10 μM hemin, 2 mM Ca^{2+}, 5 mM Mg^{2+}), while "HRP (Optimised)" represents the final optimised condition (15 μM hemin, 0.5 mM Ca^{2+}, 2 mM Mg^{2+}). All conditions contain disulphide bond enhancers (DSB).

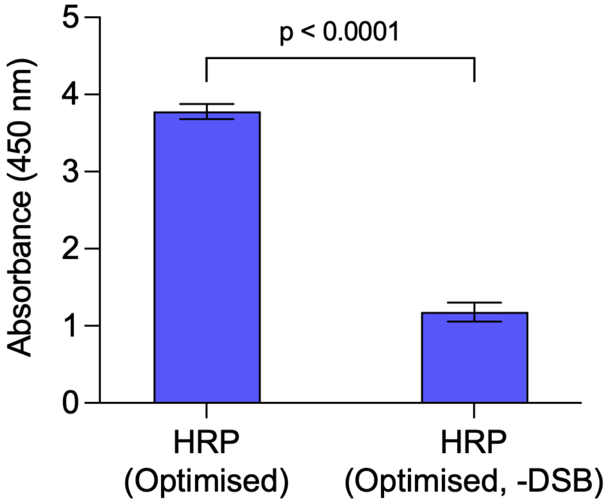

Figure S3: **Effect of disulfide bond enhancers (DSB) on HRP expression in the cell-free system.** HRP activity was measured under optimised conditions (20 °C, 15 μM hemin, 0.5 mM Ca^{2+}, 2 mM Mg^{2+}) in the presence of DSB "HRP (Optimised)" or absence of DSB "HRP (Optimised, -DSB)". Data for the HRP (Optimised) condition are replotted from Figure S2. Activity was quantified by spectrophotometric detection of TMB oxidation following a 500-fold dilution of reaction products. Exclusion of DSB resulted in significantly lower HRP activity, indicating that it is beneficial to functional HRP expression

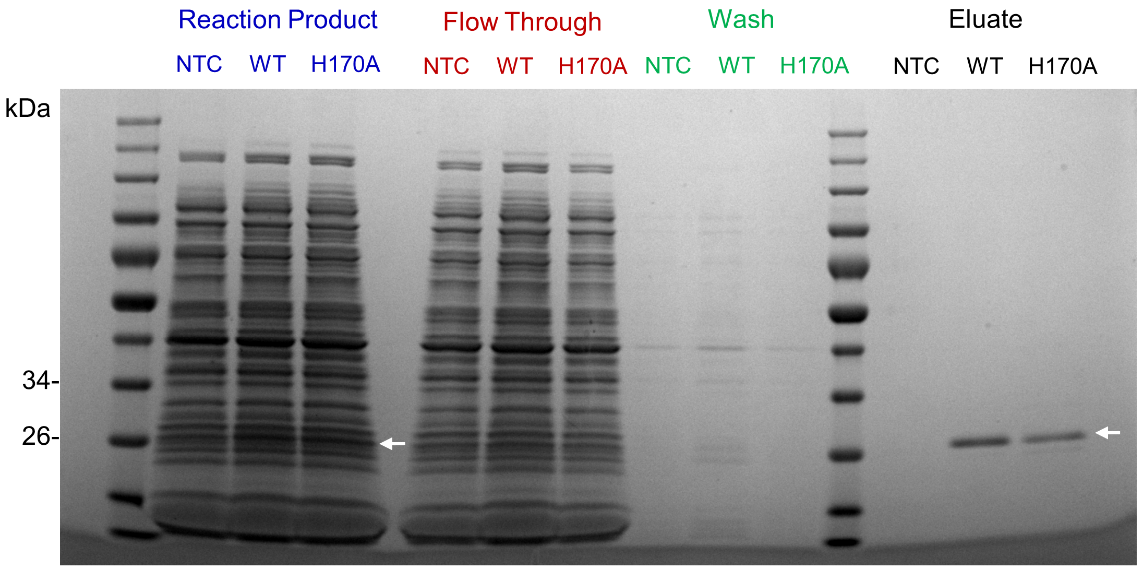

Figure S4: **SDS–PAGE analysis of cell-free expressed products and His-tag purified proteins.** Reaction products, flow-through, wash fractions, and eluted proteins were resolved by SDS–PAGE and stained with Coomassie blue. Lanes correspond to no-template control (NTC), wild-type HRP (WT), and H170A mutant, as indicated. The expected band for WT and H170A (∼32 kDa, arrows) is visible in the reaction products and can be clearly seen in the elution fractions after His-tag purification, confirming successful translation of both proteins in the cell-free system.

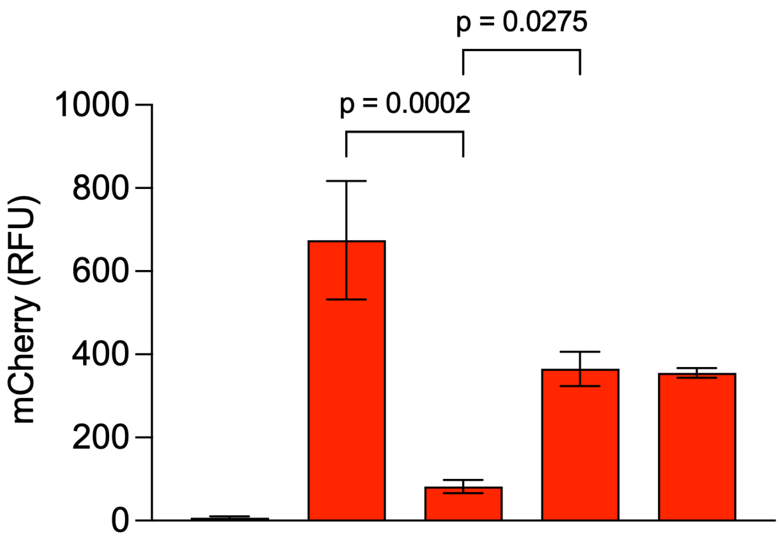

T7-TetO-mCherry	-	+	+	+	+
TetR repressor	-	-	+	+	-
aTc activator	-	-	-	+	+

Figure S5: **Optimization of a tetracycline-responsive genetic circuit using mCherry as reporter.** Fluorescence mCherry output was measured under different genetic circuit conditions to assess the requirement for reporter DNA, TetR repressor, and aTc activator (n = 3 replicates for each condition). Inclusion of aTc caused a 4.5-fold induction of mCherry expression (+DNA/+TetR/+aTc condition compared to +DNA/+TetR/-aTc condition). Reactions were performed with 10 nM T7–TetO mCherry DNA, 100 nM TetR protein, and 1 μM aTc in a total reaction volume of 10 μL, as indicated in the table below the graph.

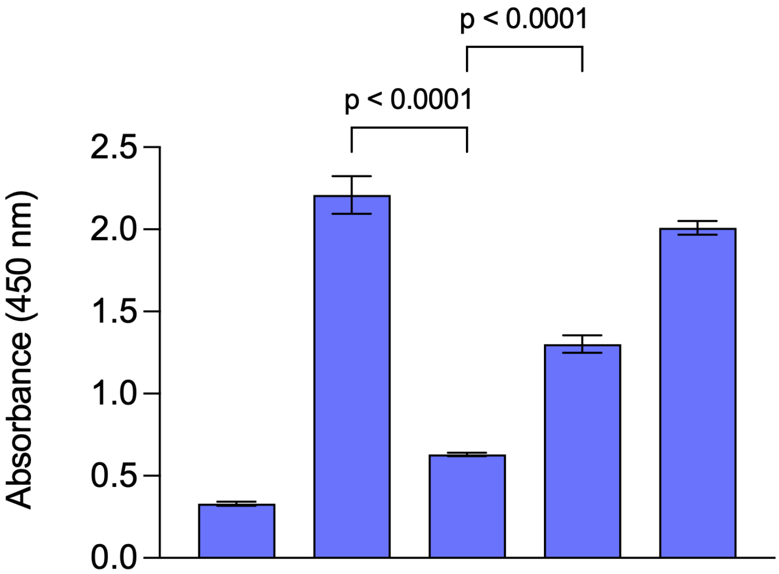

T7-TetO-HRP	-	+	+	+	+
TetR repressor	-	-	+	+	-
aTc activator	-	-	-	+	+

Figure S6: **Implementation of a tetracycline-responsive genetic circuit using HRP as reporter.** HRP activity was quantified under different genetic circuit conditions by spectrophotometric detection of TMB oxidation (n = 3 replicates for each condition). Inclusion of aTc caused a 2.1-fold induction of HRP expression (+DNA/+TetR/+aTc condition compared to +DNA/+TetR/-aTc condition). Reactions contained 10 nM T7–TetO HRP DNA, 100 nM TetR protein, and 1 μM aTc activator in a total reaction volume of 10 μL, as indicated in the table below the graph.

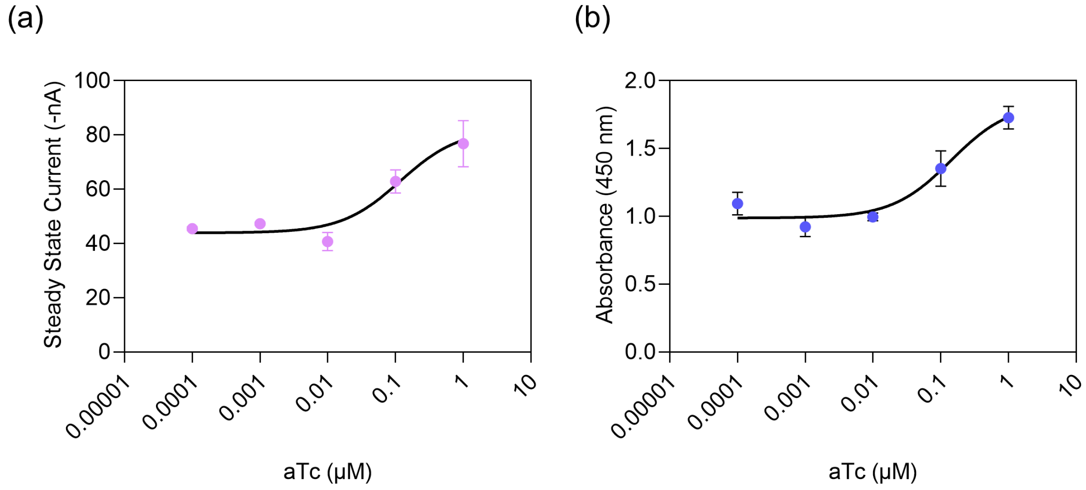

Figure S7: **Dose-response characterisation of the aTc-inducible sensor using HRP as a reporter.** (a) Electrochemical response showing steady-state current as a function of increasing aTc concentration. (b) Colorimetric response measured as absorbance following TMB oxidation. Both datasets exhibit a sigmoidal response, indicating dose-dependent induction of HRP expression with increasing aTc concentrations.

2 Supplementary Tables

Table S1: Primers used for linear PCR amplification.

**Primer**	**Sequence (5'–3')**
Forward	TGCTGCAAGGCGATTAAGTT
Reverse	TGTAGGCATAGGCTTGGTTA

Table S2: Cell-free TX-TL reaction mixture compositions. Concentrations and components that are specific to HRP or mCherry reactions are indicated in brackets.

**Component**	**Final concentration**	**Units**
E. coli extract	10	mg/mL
Energy solution	1	×
Amino acid solution	1	×
PEG 8000	2	%
Magnesium glutamate	2 (HRP) / 8 (mCherry)	mM
Potassium glutamate	100	mM
Maltose	15	mM
DTT	1.5	mM
Chi-6	5	μM
DNA Template	10	nM
Hemin	15 (HRP) / 0 (mCherry)	μM
Calcium acetate	0.5 (HRP) / 0 (mCherry)	mM
DSB 1	1 (HRP) / 0 (mCherry)	×
DSB 2	1 (HRP) / 0 (mCherry)	×

Table S3: Energy solution composition.

	**Concentration**		
**Component**	**14× (stock)**	**1× (final)**	**Units**
HEPES pH 8	700	50	mM
ATP	21	1.5	mM
GTP	21	1.5	mM
CTP	12.6	0.9	mM
UTP	12.6	0.9	mM
tRNA	2.8	0.2	mg/ml
CoA	3.64	0.26	mM
NAD	4.62	0.33	mM
cAMP	10.5	0.75	mM
Folinic acid	0.95	0.068	mM
Spermidine	14	1	mM
3-PGA	420	30	mM

Table S4: Amino acid solution composition (components from RTS Amino Acid Sampler kit, Biotechrabbit).

**Component**	**Concentration** $4\times$ **(stock)**	**Concentration** $1\times$ **(final)**	**Units**
Ala	6	1.5	mM
Arg	6	1.5	mM
Asn	6	1.5	mM
Asp	6	1.5	mM
Gln	6	1.5	mM
Glu	6	1.5	mM
Gly	6	1.5	mM
His	6	1.5	mM
Ile	6	1.5	mM
Lys	6	1.5	mM
Met	6	1.5	mM
Phe	6	1.5	mM
Pro	6	1.5	mM
Ser	6	1.5	mM
Thr	6	1.5	mM
Val	6	1.5	mM
Trp	6	1.5	mM
Tyr	6	1.5	mM
Leu	5	1.25	mM
Cys	6	1.5	mM
